# Macrophages and Their Organ Locations Shape Each Other in Development and Homeostasis – A *Drosophila* Perspective

**DOI:** 10.3389/fcell.2021.630272

**Published:** 2021-03-11

**Authors:** Anjeli Mase, Jordan Augsburger, Katja Brückner

**Affiliations:** ^1^Department of Cell and Tissue Biology, University of California, San Francisco, San Francisco, CA, United States; ^2^Eli and Edythe Broad Center of Regeneration Medicine and Stem Cell Research, University of California, San Francisco, San Francisco, CA, United States; ^3^Cardiovascular Research Institute, University of California, San Francisco, San Francisco, CA, United States

**Keywords:** *Drosophila melanogaster*, macrophage, plasmatocyte, hemocyte, organ microenvironment, regeneration, development, homeostasis

## Abstract

Across the animal kingdom, macrophages are known for their functions in innate immunity, but they also play key roles in development and homeostasis. Recent insights from single cell profiling and other approaches in the invertebrate model organism *Drosophila melanogaster* reveal substantial diversity among *Drosophila* macrophages (plasmatocytes). Together with vertebrate studies that show genuine expression signatures of macrophages based on their organ microenvironments, it is expected that *Drosophila* macrophage functional diversity is shaped by their anatomical locations and systemic conditions. *In vivo* evidence for diverse macrophage functions has already been well established by *Drosophila* genetics: *Drosophila* macrophages play key roles in various aspects of development and organogenesis, including embryogenesis and development of the nervous, digestive, and reproductive systems. Macrophages further maintain homeostasis in various organ systems and promote regeneration following organ damage and injury. The interdependence and interplay of tissues and their local macrophage populations in *Drosophila* have implications for understanding principles of organ development and homeostasis in a wide range of species.

## Introduction

Macrophages have a wide range of functions across species. While best known for their roles in innate immunity, macrophages also perform vital tissue-specific roles in development and homeostasis (Gold and Brückner, 2015; Okabe and Medzhitov, 2016). At the same time, macrophages are defined by their local microenvironments (Lavin et al., 2015). In this review, we discuss these underappreciated dual ways that macrophages and their microenvironment shape one another, focusing on insights from the invertebrate model organism *Drosophila melanogaster*.

The *Drosophila* blood cell system closely parallels the hematopoietic system of vertebrates both developmentally and functionally, making it an especially apt model for studying macrophage development, heterogeneity, and function (Hartenstein, 2006; Gold and Brückner, 2014, 2015; Banerjee et al., 2019). Since the 1970s, the concept of the mononuclear macrophage system dominated the vertebrate field, proposing that hematopoietic progenitors of the bone marrow give rise to monocytes, which are the source of all macrophages as they enter the tissues (van Furth et al., 1972; Dzierzak and Speck, 2008; Morrison and Scadden, 2014). However, over the last decade or more, this view has been dismissed in favor of a new model of two independent lineages of macrophages (Frame et al., 2013; Lavin et al., 2015; Ginhoux et al., 2016; Perdiguero and Geissmann, 2016). Based on modern genetic lineage tracing, an independent lineage of blood cells gives rise to the majority of tissue-resident macrophages in vertebrates. This independent lineage derives from erythro-myeloid progenitors that originate in the embryonic yolk sac and mature in the fetal liver, and subsequently colonize organs throughout the body, giving rise to local macrophage populations such as the microglia of the brain, Langerhans cells of the skin, Kupffer cells of the liver, and resident macrophages of the lung (Herbomel et al., 2001; Merad et al., 2002; Ajami et al., 2007; Geissmann et al., 2010; Hoeffel et al., 2012; Schulz et al., 2012; Davies et al., 2013; Hashimoto et al., 2013; Sieweke and Allen, 2013; Gomez Perdiguero et al., 2015). In some, but not all, organs this independent lineage of tissue macrophages is complemented by macrophages of the monocyte lineage (Davies et al., 2013; Frame et al., 2013; Sieweke and Allen, 2013; Lavin et al., 2015; Ginhoux et al., 2016; Perdiguero and Geissmann, 2016).

Interestingly, much like vertebrates, *Drosophila* also has two independent lineages of blood cells, or hemocytes:

(1) The embryonic/resident lineage, which parallels the vertebrate erythro-myeloid progenitor lineage of tissue macrophages, is based on self-renewing differentiated macrophages (Makhijani et al., 2011; Davies et al., 2013; Gold and Brückner, 2014, 2015; Ratheesh et al., 2015; Banerjee et al., 2019). Hemocytes of this lineage arise in the embryonic head mesoderm, quickly differentiate into macrophage-like plasmatocytes, migrate throughout the embryo in stereotyped routes (Tepass et al., 1994; Siekhaus et al., 2010), and then colonize organ and tissue microenvironments in the larva where they proliferate over time (Gold and Brückner, 2014, 2015). Examples include the prominent tissue-resident clusters of hemocytes in segmentally repeated epidermal-muscular pockets (hematopoietic pockets), and resident hemocytes at the proventriculus of the gastrointestinal system (Zaidman-Rémy et al., 2012). Homing and adhesion of hemocytes to these sites depends on active sensory neurons of the hematopoietic pockets and their expression of the Transforming Growth Factor-β (TGF-β) family ligand Activin-β (Actβ) and other predicted factors (Makhijani et al., 2011, 2017). Neuron signals may also play a role in the localization of hemocytes at the proventriculus (Cognigni et al., 2011). In hemocytes, actin cytoskeleton regulators such as Rho1 and Rac appear to be required for their tissue localization and adhesion (Williams, 2006; Makhijani et al., 2011). Likewise, Nimrod family transmembrane receptors such as Nimrod C1 (NimC1) and Eater, expressed on plasmatocytes, play roles in adhesion (Bretscher et al., 2015; Melcarne et al., 2019), the latter through interaction with the collagen XV/XVIII ortholog Multiplexin in the basement membrane of tissues (Csordás et al., 2020). Hemocyte adhesion is negatively regulated by factors from other tissues such as NimB5, secreted from the fat body upon nutrient starvation, driving hemocyte release into circulation (Ramond et al., 2020b). Resident hemocytes also lose adhesion and enter circulation upon various immune challenges, or changes in cell signaling (Williams, 2006; Stofanko et al., 2008; Markus et al., 2009), while wounds induce local adhesion of circulating hemocytes (Babcock et al., 2008). However, under unchallenged conditions, in the first and second instar larva, the vast majority of hemocytes are resident (Makhijani et al., 2011; Petraki et al., 2015). Starting in the late second to early third instar, an increasing number of hemocytes enter circulation (Markus et al., 2009; Makhijani et al., 2011; Petraki et al., 2015), establishing a steady state exchange with various resident locations (Welman et al., 2010; Makhijani et al., 2011; Makhijani and Brückner, 2012).

(2) The lymph gland lineage, which is based on progenitors, parallels the vertebrate lineage of hematopoietic stem and progenitor cells (Jung, 2005; Krzemien et al., 2010a, b; Gold and Brückner, 2014; Banerjee et al., 2019). Developmentally, the lymph gland originates from the cardiogenic mesoderm of the embryo, echoing the emergence of hematopoietic stem cells from the endothelium of the aorta in vertebrates (Hartenstein, 2006; Gold and Brückner, 2014, 2015; Banerjee et al., 2019). Blood progenitors of the lymph gland proliferate in the embryo and the larval stages, and only start in the mid-second instar to differentiate into plasmatocytes and other immune cell types (Jung, 2005; Krzemien et al., 2010a; Gold and Brückner, 2015; Banerjee et al., 2019). In addition, differentiated plasmatocytes proliferate to a certain extent, in particular in third instar larvae (Jung, 2005; Banerjee et al., 2019). Immune assaults and environmental challenges accelerate the differentiation of lymph gland progenitors and the release of differentiated plasmatocytes and other immune cells into circulation (Sorrentino et al., 2002; Crozatier et al., 2004; Márkus et al., 2005; Owusu-Ansah and Banerjee, 2009; Shim et al., 2013; Letourneau et al., 2016; Banerjee et al., 2019). Likewise, dysregulation of various major signaling pathways that usually tightly control normal lymph gland development can result in premature, or precocious, differentiation, including signaling by Notch (N), Hedgehog (Hh), Wingless (Wg), the Bone Morphogenetic Protein (BMP) Decapentaplegic (Dpp), receptor tyrosine kinases such as the PDGFR/VEGFR-related Receptor (PVR) and Fibroblast Growth Factor Receptor (FGFR), Hippo, JAK/STAT, NFκB- related Toll signaling and transcriptional regulators such as the zinc finger transcription factor Zfrp8 and the GATA factor Pannier (Qiu et al., 1998; Myrick and Dearolf, 2000; Lebestky et al., 2003; Crozatier et al., 2004; Mandal et al., 2007; Minakhina et al., 2007, 2011; Sinenko et al., 2009; Pennetier et al., 2012; Dragojlovic-Munther and Martinez-Agosto, 2013; Ferguson and Martinez-Agosto, 2014; Milton et al., 2014; Destalminil-Letourneau et al., 2021). In contrast, under unchallenged conditions, the lymph gland disintegrates and releases all of its hemocytes at the beginning of metamorphosis (Grigorian et al., 2011).

The two hemocyte lineages persist into the adult animal, with the embryonic lineage contributing the major part of immune cells, at least under unchallenged conditions (Holz et al., 2003; Sanchez Bosch et al., 2019). No significant new blood cell production has been detected in the adult, even under conditions of immune challenge (Sanchez Bosch et al., 2019), and a decline in both hemocyte number and phagocytic activity has been documented as adult flies age (Mackenzie et al., 2011; Horn et al., 2014). Both hemocyte lineages give rise to common cell types: plasmatocytes (>90% of immune cells at most developmental stages), which are analogous to vertebrate macrophages and function as the primary phagocytic cells in *Drosophila*; crystal cells (∼5% of immune cells), which function in clotting and wound healing through prophenoloxidase (PPO)-mediated melanization; and lamellocytes, stress- or immune challenge-induced cells involved in encapsulation, analogous to granuloma formation in vertebrates (Gold and Brückner, 2014, 2015; Banerjee et al., 2019).

**FIGURE 1:**
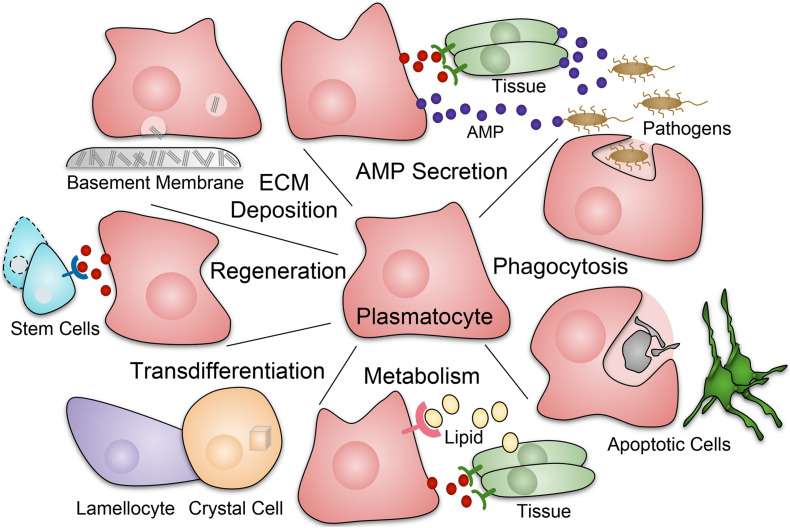
Roles of macrophage-like plasmatocytes in *Drosophila*. Plasmatocytes (red) perform a diverse array of functions during development, homeostasis, injury, and infection. Responses include phagocytosis of pathogens and apoptotic cells; production of AMPs (antimicrobial peptides) and inflammatory mediators; production and deposition of ECM (extracellular matrix) components such as collagen that are often part of the basement membrane; tissue repair and regeneration, including stimulation of stem cell function; roles in metabolic homeostasis including uptake of lipids and secretion of metabolic mediators. In addition, at least some *Drosophila* plasmatocytes have plasticity to transdifferentiate into other hemocyte types, specifically crystal cells and, upon immune challenge, lamellocytes.

Across species, macrophages have many important functions during development and homeostasis (Figure 1). Macrophages play vital roles in phagocytosis of pathogens and apoptotic cells, through scavenger receptors such as Croqumort (Crq), and Nimrod-domain (NIM) containing receptors including Eater, Nimrod C1 (NimC1), Draper (Drpr), and Six-microns-under (Simu) (Franc, 1999; Manaka et al., 2004; Kocks et al., 2005; MacDonald et al., 2006; Kurucz et al., 2007; Kurant et al., 2008; Krzemien et al., 2010b; Melcarne et al., 2019; Roddie et al., 2019). Related to this, macrophages participate in wound healing (Stramer et al., 2005; Babcock et al., 2008; Pastor-Pareja et al., 2008; Koh and DiPietro, 2011; Wang et al., 2014). They play a central role in innate immunity, producing antimicrobial and pro-inflammatory mediators (Lemaitre and Hoffmann, 2007; Lazzaro, 2008; Buchon et al., 2014). In addition, macrophages have homeostatic functions such as regulation of dietary stress (Woodcock et al., 2015) and detection and regulation of the metabolic state (Parupalli et al., 2020). *Drosophila* macrophages also produce and deposit extracellular matrix (ECM) components (Fessler and Fessler, 1989; Wood and Jacinto, 2007) such as Collagen IV, Laminin, Perlecan, and Peroxidasin, an ECM-associated peroxidase, as they migrate along surfaces and reside in specific anatomical locations (Nelson et al., 1994; Bunt et al., 2010; Martinek et al., 2011; Van De Bor et al., 2015; Matsubayashi et al., 2017; Sánchez-Sánchez et al., 2017). Moreover, *Drosophila* macrophages regulate stem cells and other tissue-specific cell populations, often through localized secretion of signals such as cytokines of the Unpaired (Upd) family, which signal through the receptor Domeless (Dome) and the JAK/STAT pathway (Hopscotch and Stat2E in *Drosophila*), promoting proliferation and differentiation of target tissues (Chakrabarti et al., 2016; Guillou et al., 2016). In *Drosophila*, at least some macrophage-like plasmatocytes have the plasticity to give rise to crystal cells (Bretscher et al., 2015; Leitão and Sucena, 2015; Corcoran et al., 2020) and, upon immune challenge, lamellocytes (Markus et al., 2009; Anderl et al., 2016).

## New Insights Into Macrophage Diversity

A recent body of research suggests that not all macrophages are equal, rather they can be categorized into phenotypically and functionally unique subpopulations. Single cell RNA sequencing and functional studies in *Drosophila* identify transcriptionally and functionally distinct clusters of plasmatocytes, which are modulated by developmental time, lineage, injury, and infection status (Cattenoz et al., 2020; Cho et al., 2020; Coates et al., 2020; Ramond et al., 2020a; Tattikota et al., 2020). Vertebrate single cell studies identify similar heterogeneity among macrophages, modulated by developmental stage and lineage (Gordon and Taylor, 2005; Martinez et al., 2006; Paul et al., 2015; Lin et al., 2019; Lantz et al., 2020). Under pathologic conditions, macrophages may take on a spectrum of activation states, mirrored by their transcriptional profiles, dependent on disease severity (Lin et al., 2019; Mould et al., 2019; Weinstock et al., 2019; Lantz et al., 2020). Additionally, through analyses of enhancer landscapes and tissue-specific single cell RNA sequencing, it has become clear that macrophage subpopulations of organs including the liver, spleen, lung, peritoneal cavity, colon, small intestine, brain, and kidney, are shaped by their tissue of residence in vertebrate models (Gosselin et al., 2014; Lavin et al., 2014; MacParland et al., 2018; Mould et al., 2019; Van Hove et al., 2019; Zimmerman et al., 2019). In *Drosophila*, it is expected that heterogeneity of macrophages stems from the complex interactions between these cells and their microenvironment. A recent sequencing study identified cross-species markers between *Drosophila* and vertebrate macrophages, although their functional significance remains to be determined (Fu et al., 2020).

In addition to parallels in the local regulation of macrophages, *Drosophila* and vertebrates rely on conserved systemic signaling that regulates macrophages. Vertebrate macrophages express colony stimulating factor 1 receptor (CSF-1R), a receptor tyrosine kinase (RTK) of the family of Platelet-Derived Growth Factor Receptors and Vascular Endothelial Growth Factor Receptors (PDGFRs and VEGFRs) (Lemmon and Schlessinger, 2010). Vertebrate CSF-1R is activated by colony-stimulating factor-1 (CSF-1) and interleukin-34 (IL-34), promoting proliferation, differentiation, survival, chemotactic migration and differentiation of macrophages during development, homeostasis, and innate immunity (Stanley and Chitu, 2014). Similarly in *Drosophila*, the molecularly conserved ortholog PDGFR/VEGFR-related Receptor (PVR) is expressed in hemocytes. PVR recognizes PDGF/VEGF related factors Pvf1, Pvf2, and Pvf3, and is essential for trophic survival, proliferation, plasmatocyte activation, and some aspects of chemotactic migration (Munier et al., 2002; Brückner et al., 2004; Wood et al., 2006; Wood and Jacinto, 2007; Kelsey et al., 2012; Parsons and Foley, 2013; Sopko et al., 2015).

Despite the apparent necessity of macrophages in development and homeostasis, ablation studies suggest that hemocytes are not essential for survival in *Drosophila* (Braun et al., 1998; Charroux and Royet, 2009; Defaye et al., 2009; Arefin et al., 2015). However, plasmatocytes and PVR expression are fundamental to embryonic development, as they promote the essential process of central nervous system (CNS) condensation (Zhou et al., 1995; Sears, 2003; Olofsson and Page, 2005; Defaye et al., 2009; Evans et al., 2010). Lack of macrophages is seemingly compatible with larval and pupal development, although it causes a shift in immune effector pathways – specifically, induction of the Toll pathway and repression of the Imd pathway – which leads to a proinflammatory state and aberrant leg development, in turn resulting in reduced likelihood of eclosion (Arefin et al., 2015). In adult *Drosophila*, lack of macrophages increases susceptibility to bacterial infection (Braun et al., 1998; Charroux and Royet, 2009; Defaye et al., 2009; Arefin et al., 2015), demonstrating their immune functions and role as sentinels of infection that induce antimicrobial peptide (AMP) gene expression in other tissues (Sanchez Bosch et al., 2019).

**FIGURE 2:**
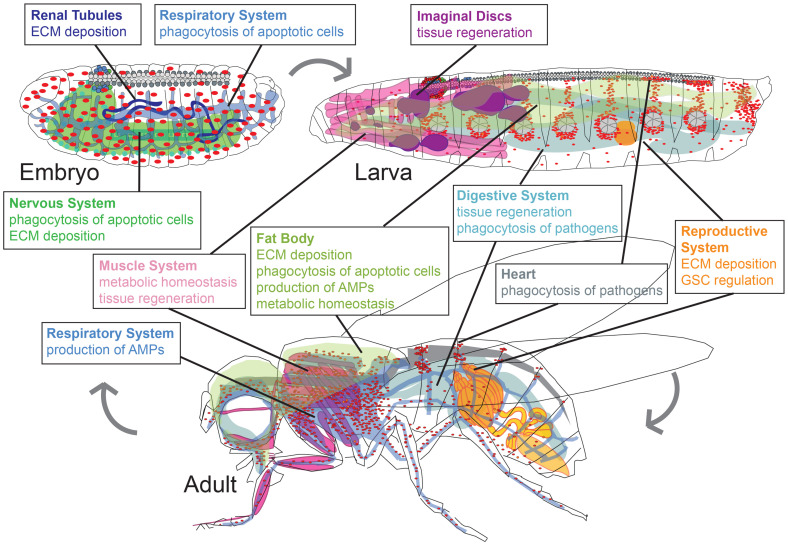
*Drosophila* macrophages play life stage and tissue-specific roles in development, homeostasis, and infection. Major roles of plasmatocytes (macrophages, red) during developmental stages of embryo, larva, and adult (pupal stage is omitted). Organ systems regulated by macrophages are renal tubules (dark blue), respiratory system (light blue), nervous system (green), fat body (olive green), muscle system (pink), imaginal discs (purple), digestive system (teal), reproductive system (orange), heart (gray). Organ shapes adapted from Hartenstein (1995).

It is clear that plasmatocytes are a diverse population of cells that modulate a wide variety of processes during development. Genetic studies in *Drosophila* have provided broad evidence of tissue-specific macrophage function. How do macrophages and their microenvironment shape one another in different organ systems? We address this question in the following paragraphs and Figure 2.

## Nervous System

The *Drosophila* central nervous system consists of the brain and ventral nerve cord, and the peripheral nervous system includes sensory and motor neurons (Landgraf and Thor, 2006; Sánchez-Soriano et al., 2007; Hartenstein et al., 2008; Singhania and Grueber, 2014; Schirmeier et al., 2016; Doe, 2017; Li et al., 2018; Sugie et al., 2018; Akin and Zipursky, 2020). Major roles of macrophages in the nervous system, together with glia, are the phagocytic removal of apoptotic cells and the production of ECM (Zheng et al., 2017; Bittern et al., 2020; Hilu-Dadia and Kurant, 2020). Hemocytes invade the posterior end of the embryo during the germband extended stage and subsequently disperse throughout the embryo by migration, also entering the ventral nerve cord (VNC) (Tepass et al., 1994; Brückner et al., 2004). Some aspects of this migration, in particular invasion of the posterior end and migration along the VNC are mediated by PVR (Brückner et al., 2004; Wood et al., 2006), although PVR is primarily required for anti-apoptotic survival of hemocytes (Brückner et al., 2004). In the VNC, a significant amount of programmed cell death takes place in various cell types from the early stages of CNS formation to the end of embryogenesis (Abrams et al., 1993; White et al., 1994; Sonnenfeld and Jacobs, 1995; Zhou et al., 1995; Hidalgo et al., 2001; Peterson et al., 2002; Lundell, 2003; Miguel-Aliaga, 2004; Karcavich and Doe, 2005; Rogulja-Ortmann et al., 2007). Hemocytes phagocytose apoptotic bodies, opening up spaces and allowing for condensation of the nervous system (Olofsson and Page, 2005; Evans et al., 2010). Inhibition of hemocyte development or function causes mispositioning of glia, which, in turn, results in CNS axon scaffold and patterning defects (Sears, 2003); this phenotype is also observed when either *PVR* or *Crq* are RNAi silenced in hemocytes (Sears, 2003). CNS axon scaffold malformation forms a physical barrier to hemocyte migration along the VNC (Evans et al., 2010). When hemocytes cannot migrate, VNC condensation was proposed to also be disrupted due to a lack of ECM deposition by the migrating hemocytes (Olofsson and Page, 2005; Evans et al., 2010). Consistent with this, hemocytes deficient in *laminin B1 (LanB1)* exhibit slower migration along the ventral nerve cord (VNC) and defects in VNC condensation (Sánchez-Sánchez et al., 2017).

As development proceeds, in the larva, and especially during metamorphosis and in the adult, functions of hemocytes are more predominantly adopted by glia. In particular, glia mediate phagocytosis of dead cells and neuron fragments during axonal and dendrite pruning, and following injury (Sonnenfeld and Jacobs, 1995; Watts et al., 2004; Kurant, 2011; Bittern et al., 2020; Furusawa and Emoto, 2020; Hilu-Dadia and Kurant, 2020). Hemocytes and glia show molecular parallels regarding their phagocytic receptors such as Simu and Drpr (MacDonald et al., 2006; Kurant et al., 2008; Shklyar et al., 2014; Evans et al., 2015; Weavers et al., 2016; Shlyakhover et al., 2018; Davidson and Wood, 2020), and their mutual dependence on the transcription factors glial cells missing (gcm) and glial cells missing 2 (gcm2), during embryonic development (Bernardoni et al., 1997; Alfonso and Jones, 2002; Trébuchet et al., 2019).

Plasmatocytes also play roles in the development and homeostasis of the peripheral nervous system (PNS). During larval development, macrophages were proposed to function in neuronal pruning by severing destabilized dendritic branches and engulfing neuronal debris (Williams, 2005). More recently, however, it has been shown that non-traditional phagocytes, including glia and epidermal cells, play more central roles in neuronal pruning during development (Han et al., 2014). Macrophages may also have other roles in PNS development: a study suggested that hemocytes may promote aspects of glial cell biology necessary for peripheral nerve elongation (Pandey et al., 2011).

Conversely, the peripheral nervous system (PNS) can shape its resident macrophages and other hemocytes. Hemocytes associate with sensory neurons of the PNS in segmentally repeated hematopoietic pockets of the larval body wall (Makhijani et al., 2011; Makhijani and Brückner, 2012). These sensory neurons detect a variety of environmental and internal cues such as mechanical inputs, chemical stimuli, temperature, and light (Tracey et al., 2003; Hughes and Thomas, 2007; Song et al., 2007; Xiang et al., 2010; Han et al., 2014) and serve as a microenvironment for macrophages and other hemocytes (Makhijani et al., 2011, 2017; Makhijani and Brückner, 2012; Corcoran et al., 2020). Within the microenvironments, neurons promote macrophage survival (Makhijani et al., 2011) through Dscam1 expression (Ouyang et al., 2020), and proliferation and localization by the expression of Actβ (Makhijani et al., 2017). Moreover, a specific set of caudal sensory neurons promotes transdifferentiation of plasmatocytes into crystal cells in the presence of oxygen, providing evidence that environmental inputs to the sensory nervous system can impact hematopoietic processes (Corcoran et al., 2020).

## Digestive System

The digestive system of *Drosophila* is maintained throughout all developmental stages based on intestinal stem cell (ISC) proliferation and differentiation (Murakami et al., 1999; Micchelli and Perrimon, 2006; Ohlstein and Spradling, 2006; Lemaitre and Miguel-Aliaga, 2013). Macrophages and other hemocytes form aggregates in folds of the intestine: at all developmental stages, they are enriched at the proventriculus, a structure where the esophagus, crop, and midgut converge (Tepass et al., 1994; Lebestky et al., 2000; Brückner et al., 2004; Charroux and Royet, 2009; Zaidman-Rémy et al., 2012). Hemocytes at the proventriculus are regulated by phosphoinositide 3-kinase (PI3K): PI3K signaling decreases hemocyte adhesion at the proventriculus, although it does not interfere with initial recruitment (Zaidman-Rémy et al., 2012). Hemocyte localization and responses may be further regulated by the innervation of the proventriculus (Cognigni et al., 2011), similar to hemocyte dependence on active sensory neurons in the hematopoietic pockets (Makhijani et al., 2011, 2017; Gold and Brückner, 2014, 2015). Macrophages are also enriched in clusters at the midgut, especially upon damage or infection (Ayyaz et al., 2015), and it has been debated whether some hemocytes are inserted in the midgut epithelium (Charroux and Royet, 2009; Zaidman-Rémy et al., 2012).

The macrophages of the intestine play important roles in innate immunity, maintaining homeostasis of gut microbiota both under basal conditions and after pathogen ingestion via the secretion of AMPs and phagocytosis (Nehme et al., 2007; Charroux and Royet, 2009; Ayyaz et al., 2015; Bonfini et al., 2016; Guillou et al., 2016). In addition, under conditions of tissue damage, inflammation, and infection, local and systemic macrophages function to promote and control tissue regeneration of the intestine (Takeishi et al., 2013; Ayyaz et al., 2015; Chakrabarti et al., 2016). Septic injury triggers upregulation of Upd ligands in hemocytes (Pastor-Pareja et al., 2008; Chakrabarti et al., 2016), inducing systemic changes including intestinal stem cell activation via JAK/STAT signaling (Chakrabarti et al., 2016; Guillou et al., 2016); this mechanism plays a role in many situations of gut regeneration and homeostasis (Jiang et al., 2009). Upon gut damage by oxidative stress or oral infection, local hemocytes produce the BMP Dpp, inducing proliferation of ISCs through activation of the signal transducer dSmad2, followed by signaling through the signal transducer Mothers against Dpp (Mad) that restores ISC quiescence (Ayyaz et al., 2015). This pathway also maintains normal gut homeostasis and limits ISC proliferation (Guo et al., 2013; Zhou et al., 2015). As the fly ages, repeated Dpp secretion by hemocytes can lead to dysregulated dSmad2 activity, resulting in dysplasia as ISCs over-proliferate, an intriguing model for colon cancer (Ayyaz et al., 2015). Similarly, in mouse models, macrophages in the colon directly promote the proliferation of epithelial progenitors as a wound response (Pull et al., 2005).

## Reproductive System

The *Drosophila* reproductive organs, the female ovary and the male testis, carry germline stem cells and produce the gametes (Fuller and Spradling, 2007). In the *Drosophila* ovary, the basement membrane, a specialized ECM underlying the basal side of epithelial cells (LeBleu et al., 2007; Yurchenco, 2011), is important for the stability and function of the gonad and the developing egg chambers (follicles) (Denef et al., 2008; Van De Bor et al., 2015). While follicular epithelial cells contribute to the basement membrane in the adult ovary (Denef et al., 2008), a population of ovarian macrophages deposits collagen IV in the larval gonad, forming a stable basement membrane that persists from the larval to the adult stage (Van De Bor et al., 2015). This basement membrane regulates the stem cell niche of the gonad by limiting diffusion of the BMP Dpp, which prevents excessive Dpp in the ovaries from triggering over-proliferation of the germline stem cells (Van De Bor et al., 2015). The gradient of Dpp is also known to be limited by heparan sulfate proteoglycans (HSPGs) (Guo et al., 2013; Zhou et al., 2015). In the absence of hemocyte-produced collagen, larvae develop malformed basement membranes, which, in turn, lead to dysregulated stem cell proliferation, ultimately resulting in decreased reproductive fitness (Wang et al., 2008; Van De Bor et al., 2015). In the male testis, macrophages were suggested to be required for the regeneration of germline stem cells from dedifferentiating spermatogonial cells, a process that may depend on JAK/STAT signaling (Varga et al., 2020). In the mammalian ovary, macrophages play various roles, although mechanistic parallels with *Drosophila* remain to be determined (Wu et al., 2004). For example, macrophages are located along the basement membrane and support follicular development during the estrous cycle (Cohen et al., 1997). Their exact role in tissue remodeling during ovulation is unclear, although mice lacking ovarian macrophages have decreased reproductive success (Cohen et al., 1997).

## Respiratory System

The *Drosophila* respiratory system consists of a tubular system of trachea that develop over the embryonic and larval stages; it is then remodeled during metamorphosis, forming the extensive air sacs of the head and thorax, as well as tubular tracheal structures in the abdomen of the adult animal (Whitten, 1957; Manning and Krasnow, 1993; Hayashi and Kondo, 2018). In the tracheal system, hemocytes exist alongside respiratory tubes and epithelia to assist with development (Hartenstein et al., 1994; Baer et al., 2010) and prevention of infection (Sanchez Bosch et al., 2019). Specifically, during embryonic development, tracheal cells at the base of the dorsal branch undergo apoptosis in response to microenvironmental cues, detach from the epithelium, and are engulfed by macrophages (Baer et al., 2010). Tracheal cells also undergo apoptosis as part of tracheal remodeling during metamorphosis, suggesting a possible role for phagocytosing macrophages at this stage of development (Chen and Krasnow, 2014). Following pupariation, macrophages of the embryonic lineage and dispersed lymph gland hemocytes (Holz et al., 2003; Grigorian et al., 2011) associate with the respiratory epithelia (air sacs) in the head and thorax of the adult animal, forming the major blood cell reservoir (Sanchez Bosch et al., 2019). Here, macrophages act as sentinels of infection, engulfing pathogens and instructing the respiratory epithelia and neighboring cells of the fat body via secretion of Upd3 to produce e.g., the AMP Drosocin (Tzou et al., 2000; Sanchez Bosch et al., 2019), which defends against bacterial infection (Lemaitre et al., 1995; Sanchez Bosch et al., 2019).

## Fat Body

The *Drosophila* fat body is a site of energy storage, detoxification and immune response that functionally parallels the vertebrate liver (Miller et al., 2002; Dionne, 2014). It forms an extensive tissue in the embryo and larva, and lines the majority of the adult cuticle and epidermis (Zhang and Xi, 2015). During larval development, macrophages contribute critically to basement membrane formation of the fat body via the deposition of SPARC, a glycoprotein involved in the assembly of collagen IV (Shahab et al., 2015). Later, during metamorphosis, larval fat body cells undergo remodeling, resulting in fat body dissociation and apoptosis (Nelliot et al., 2006; Sanchez Bosch et al., 2019). Macrophages associate with these decaying cells and are involved in the phagocytosis of the cellular debris, a process that lasts well into adulthood (Nelliot et al., 2006; Sanchez Bosch et al., 2019).

Macrophages affect many aspects of metabolic regulation, homeostasis and immunity in the fat body (Dionne, 2014). For example, when larvae are raised on a high fat diet, or exposed to parasitic wasp infestation, plasmatocytes produce excess Upd3, inducing Jak/Stat signaling in the fat body, which downregulates insulin production, decreases larval growth, and reduces lifespan (Woodcock et al., 2015; Shin et al., 2020). This mechanism is mirrored in animals on a high sucrose diet (Parupalli et al., 2020). In response to bacterial infection, hemocytes communicate to the fat body through signals including Upd3 and the Toll ligand Spätzle to induce AMP expression, in both the larva and adult (Agaisse et al., 2003; Brennan et al., 2007; Charroux and Royet, 2009; Shia et al., 2009; Honti et al., 2014; Sanchez Bosch et al., 2019). Bacterial infection also stimulates hemocytes to release ImpL2, an insulin/IGF antagonist, which induces the release of lipoproteins and carbohydrates from the fat body to fuel the immune response (Gabriela et al., 2020); in turn, hemocytes switch to aerobic glycolysis, which supports the antibacterial defense (Krejèová et al., 2019).

Conversely, fat body cells regulate hemocyte populations during instances of nutrient deprivation and immune challenge. During starvation, macrophages move from the hematopoietic pockets and other locations to infiltrate the larval fat body (Shim et al., 2012). Specifically, the fat body releases NimB5, which acts on hemocytes to downregulate adhesion and proliferation (Ramond et al., 2020b). This mechanism redirects resources to essential functions only, promoting animal survival (Ramond et al., 2020b). Under immune challenge such as parasitic wasp infestation, signals from the fat body promote hemocyte responses: Toll signaling in the fat body promotes the Toll-dependent activation of macrophages (Schmid et al., 2014). Moreover, fat body cells upregulate expression of Edin (elevated during infection), a secreted peptide that promotes an increase in macrophage number and also triggers their release from the hematopoietic pockets and other resident locations, thereby facilitating the encapsulation response (Vanha-aho et al., 2015).

## Muscle System

*Drosophila* has a complex muscle system at all stages of development (Bothe and Baylies, 2016). Macrophages and the muscular system form another axis of communication in *Drosophila.* In the adult animal, under non-inflammatory physiology, hemocytes constitutively produce Upd3, which promotes basal JAK/STAT activity in muscle cells (Kierdorf et al., 2020). When disrupting this signaling by loss of the *dome* receptor in muscles, a systemic metabolic pathology develops, characterized by hyperactivation of the kinase AKT, an insulin signaling mediator, and reduced lifespan (Kierdorf et al., 2020). While this study raises new interesting questions, the interactions between muscle, insulin signaling, metabolism, and growth have been an intense focus of investigation (Demontis and Perrimon, 2009, 2010; Kwon et al., 2015).

Molecular communication in both directions, including signaling from the muscles to macrophages, is central in establishing immune responses (Yang et al., 2015; Yang and Hultmark, 2017). In the *Drosophila* larva, muscles regulate plasmatocytes in the immune response to parasitic wasp infestation. This effect is initially triggered by the release of Upd2 and Upd3 from plasmatocytes during wasp infestation, activating the JAK/STAT pathway in muscle tissue beyond basal levels (Yang et al., 2015; Shin et al., 2020). In turn, muscle cells control the plasmatocyte response, promoting the number of plasmatocyte-derived lamellocytes and enhancing the encapsulation response (Yang et al., 2015). This effect appears to depend on altered feeding behavior and is mediated by insulin signaling via TOR (target of rapamycin) in the muscles, which indirectly enhances JAK/STAT signaling in hemocytes, driving lamellocyte formation (Yang et al., 2015; Anderl et al., 2016; Yang and Hultmark, 2017). Interestingly, larval muscles are an anatomical part of the hematopoietic pockets where plasmatocytes reside in clusters, suggesting that muscle cells are an active player of this instructive microenvironment (Makhijani et al., 2011; Makhijani and Brückner, 2012; Yang et al., 2015).

## Heart

In *Drosophila* and in insects in general, macrophages accumulate in clusters at the ostia (intake valves) of the heart (Gupta, 1979), a tubular structure running along the dorsal side of the animal (Ocorr et al., 2007). Macrophages in these clusters monitor the streaming hemolymph of the open circulatory system and fulfill immune functions, phagocytosing bacteria and foreign particles (Gupta, 1979; Elrod-Erickson et al., 2000; Dionne et al., 2003; King and Hillyer, 2012; Cevik et al., 2019). Relatively little is known about interactions between macrophages and heart tissue. In *Drosophila* third instar larvae, hemocytes from resident sites increasingly enter circulation and subsequently accumulate in clusters at the ostia and pericardial nephrocytes of the larval heart (dorsal vessel) (Frasch, 1999), forming dorsal vessel-associated clusters (Markus et al., 2009; Makhijani et al., 2011; Petraki et al., 2015; Cevik et al., 2019). Hemocytes accumulate in the ECM of the dorsal vessel, which is facilitated by the heart-specific collagen Pericardin (Cevik et al., 2019). In addition, some aspects of this accumulation may be mechanical (Petraki et al., 2015; Cevik et al., 2019). In adult *Drosophila*, hemocytes accumulate at the ostia of the heart in a mesh of ECM that likewise contains Pericardin and Laminin A (Ghosh et al., 2015; Sessions et al., 2017). One study suggested that the heart serves as a ‘hematopoietic hub’ for new hemocyte production (Ghosh et al., 2015), however, this model was disproven based on evidence of a developmental mechanism of macrophage accumulation at the heart, and the absence of any significant hematopoietic activity using multiple orthogonal approaches (Sanchez Bosch et al., 2019).

## Renal Tubules

*Drosophila* Malpighian tubules (renal tubules) are excretory organs with similarity to the vertebrate kidney; they secrete waste and maintain ionic and osmotic homeostasis (Maddrell, 1972; Denholm and Skaer, 2009). During the embryonic development of the Malpighian tubules, macrophages are attracted to these growing structures through tubule expression of PVF ligands (Bunt et al., 2010). Macrophages, in turn, secrete components of the basement membrane (Bunt et al., 2010). Collagen IV is part of this, sensitizing tubule cells to the BMP ligand Dpp, which is required to promote the outgrowth of the tubules (Bunt et al., 2010). While it is known that Dpp is secreted locally, the source remains unknown; however, in the gut, Dpp is sourced from hemocytes (Guo et al., 2013; Ayyaz et al., 2015) and hemocytes could have a similar function for the Malpighian tubules. The process of macrophage-mediated tubule elongation is conserved in mice where tissue-resident macrophages contribute to renal organogenesis (Munro and Hughes, 2017).

## Imaginal Discs

Imaginal discs are the larval precursors to the adult fly eyes, wings, legs, and other appendages (Worley et al., 2012). They develop as epithelial sacs, which serve as intriguing models to study patterning, morphogenesis, and regeneration (Hariharan and Serras, 2017). Imaginal disc damage stimulates increase in macrophages that adhere to the wound (Bryant and Fraser, 1988; McClure et al., 2008; Pastor-Pareja et al., 2008; Katsuyama and Paro, 2013). In response to UV damage to the eye imaginal disc, macrophages actively promote tissue regeneration (Kelsey et al., 2012). Specifically, damaged disc cells upregulate Shnurri (Shn), a transcriptional regulator that induces *Pvf1*, which then signals to disc-associated hemocytes to activate their macrophage-like behavior (Kelsey et al., 2012). Activated hemocytes engulf apoptotic cells in the eye disc and clear debris to limit tissue damage (Kelsey et al., 2012). The activation of macrophages in this model relies at least in part on the induction of mesencephalic astrocyte-derived neurotrophic factor (MANF) (Neves et al., 2016). MANF shifts the expression of hemocyte markers and induces expression of the *Drosophila* homolog of the mammalian M2 marker *arginase1*, suggesting a process similar to the alternative activation of macrophages in vertebrates (Neves et al., 2016). Importantly, PDGF/MANF signaling of macrophages in response to retinal damage is conserved in mammals (Neves et al., 2016). Hemocytes also trigger tissue regeneration via epithelial cell proliferation in response to reactive oxygen species (ROSs) released from damaged epithelial disc cells (Fogarty et al., 2016). In a model of apoptosis-induced proliferation (AiP), in which eye disc cells were induced to die by the pro-apoptotic gene head involution defect (hid), while apoptosis was concomitantly blocked by p35 (Ryoo et al., 2004), activity of the caspase Dronc in epithelial disc cells promotes activation of the NADPH oxidase Duox that generates extracellular ROSs (Fogarty et al., 2016). ROS release activates disc-associated macrophages and induces them to secrete the TNF (tumor necrosis factor) family ligand Eiger, which activates JNK signaling in disc cells leading to proliferation (Fogarty et al., 2016). Similar mechanisms of ROS-induced JNK signaling may apply to the regeneration of damaged wing discs, although the role of hemocytes in this context remains to be investigated (Santabárbara-Ruiz et al., 2015). One study reported hemocytes to be dispensable for the regenerative growth of aseptic wounds of wing discs or transplanted leg disc fragments under conditions of combined ablation of hemocytes and fat body (Katsuyama and Paro, 2013). However, these experiments were performed in developmentally arrested larvae fed with *erg2Δ* mutant yeast (Katsuyama and Paro, 2013) that does not provide sterols necessary for the formation of the fly hormone ecdysone (Parkin and Burnet, 1986). Ecdysone controls molting, but also stimulates hemocyte phagocytic activity and mobility, and the encapsulation response (Sorrentino et al., 2002; Regan et al., 2013; Sampson et al., 2013), which could have affected experimental outcomes (Katsuyama and Paro, 2013).

## Discussion

*Drosophila* and vertebrates share many parallels in their macrophage systems, which in both cases are based on two lineages. While the anatomical origins of tissue macrophages in *Drosophila* and vertebrates differ, there are many evolutionary parallels at the molecular, cellular, and functional level. Considering that this lineage is the predominant source of macrophages in *Drosophila* (Sanchez Bosch et al., 2019), we propose that tissue macrophages may represent the ancient mechanism of macrophage production and regulation, allowing immediate adaptation to organismal and environmental conditions. This may be particularly important in species that heavily rely on innate immunity.

The diverse functional roles of *Drosophila* macrophages predict defined subpopulations, influenced by local signals from their tissue of residence, and possibly lineage and other conditions. This resembles vertebrates, in which macrophage populations have been characterized based on their polarization, i.e., their distinct functional phenotypes, regulated by microenvironmental and systemic stimuli (Gordon and Taylor, 2005; Martinez, 2008; Lavin et al., 2015; Zhu et al., 2015; Shapouri-Moghaddam et al., 2018). Vertebrate macrophages exhibit functional plasticity to differentiate into classically activated macrophages (M1) with roles in infection, and alternatively activated macrophages (M2) active in tissue repair and anti-inflammatory responses; further subdivisions are based on their prototypical activating stimuli and functionality (Martinez, 2008; Tarique et al., 2015; Zhu et al., 2015; Shapouri-Moghaddam et al., 2018). Recent analyses suggest an even greater spectrum of activation states exceeding these classifications (Mosser and Edwards, 2008; Xue et al., 2014), and lineage also plays a role in determining the properties and activation states of macrophages (Gundra et al., 2014). Macrophages may adopt potentially distinct activation states when mediating previously unknown functions, such as the transfer of mitochondria to and from target tissues including neurons and heart cells, which promotes repair after tissue damage, and stimulates the macrophage innate immune response, respectively (Jackson et al., 2016; Brestoff et al., 2020; Nicolás-Ávila et al., 2020; Raoof et al., 2020).

Research in *Drosophila* suggests that the characteristics of macrophages are changed following priming by an immune encounter, such as phagocytosis of apoptotic cells (Weavers et al., 2016; Nonaka et al., 2017; Roddie et al., 2019; Chakrabarti and Visweswariah, 2020). This interaction leads to “immune training”, consisting of changes in intracellular signaling and the repertoire of phagocytic receptors, which can determine behavior in future encounters (Weavers et al., 2016; Nonaka et al., 2017; Roddie et al., 2019; Chakrabarti and Visweswariah, 2020). Consistent with this, a study provided molecular evidence of *Drosophila* macrophages taking on an alternatively activated (M2) status in response to local cues in tissue regeneration (Neves et al., 2016). Single cell RNA sequencing and functional studies further support the hypothesis of distinct activation states in *Drosophila* macrophages, identifying subpopulations that have differential involvement in phagocytosis, metabolic homeostasis, and the humoral AMP response (Cattenoz et al., 2020; Cho et al., 2020; Coates et al., 2020; Fu et al., 2020; Ramond et al., 2020a; Shin et al., 2020; Tattikota et al., 2020). Functional distinctions are driven by developmental stage (Cattenoz et al., 2020; Cho et al., 2020), injury, and immune challenge (Coates et al., 2020; Fu et al., 2020; Ramond et al., 2020a; Tattikota et al., 2020).

Additional research will link many of the observed cellular differences between macrophage populations with their roles in specific organ systems, as exemplified in this review. A particular gap in knowledge is how microenvironmental cues shape the molecular and phenotypic status of macrophages to adapt to their distinct tasks, and how interactions between immune cell types and lineages may affect their response. In vertebrates, organ microenvironments regulate tissue macrophages through local production of CSF1, IL-34, and paracrine and autocrine TGF-β (Lavin et al., 2015). However, findings from *Drosophila* (Makhijani et al., 2011, 2017; Gold and Brückner, 2014, 2015; Corcoran et al., 2020) suggest the existence of more elaborate regulatory systems also in vertebrates, comprising, e.g., peripheral innervation or cell based environmental sensors that regulate local tissue macrophage populations through molecular signals. Understanding cellular and molecular principles of organ-macrophage communication in *Drosophila* will further broaden our insights into vertebrate macrophage systems, and contribute to approaches that harness the power of macrophages in regenerative medicine and immunology.

## Author Contributions

AM and JA wrote the text and designed the figures. KB revised the text and figures, designed figure elements, and coordinated the content of the review. All authors contributed to the article and approved the submitted version.

## Conflict of Interest

The authors declare that the research was conducted in the absence of any commercial or financial relationships that could be construed as a potential conflict of interest.
